# “*This course has made it easier for me to embrace myself and my child*”: A qualitative study of an adapted mindfulness-based stress reduction course for pregnant women with psychosocial vulnerabilities

**DOI:** 10.1371/journal.pmen.0000412

**Published:** 2025-11-07

**Authors:** Luna Berg Pedersen, Nanna Bjerg Ramsdal, Lone Overby Fjorback, Sophie Lykkegaard Ravn, Michelle Kolls, Sine Skovbjerg

**Affiliations:** 1 Danish Centre for Mindfulness, Department of Clinical Medicine, Aarhus University, Aarhus, Denmark; 2 Department of Psychology, Aarhus University, Aarhus, Denmark; 3 Department of Psychology, University of Southern Denmark, Odense, Denmark; 4 Department of Obstetrics and Gynaecology, Copenhagen University Hospital, Hvidovre, Denmark; Uganda Martyrs University, UGANDA

## Abstract

Mental disorders are commonly encountered in pregnancy complications, and women with a preconception history of mental disorders or other psychosocial vulnerabilities are at increased risk. Mental disorders, such as depression, can persist postnatal and adversely affect mother-infant bonding and infant development. Consequently, it is important to address prenatal mental health. The aim of this qualitative study was to explore the impact of an adapted Mindfulness-Based Stress Reduction (MBSR) course for pregnant women with psychosocial vulnerabilities as a method to support transition into motherhood. This study was conducted in collaboration with the Danish Centre for Mindfulness, Aarhus University, and an obstetric ambulatory clinic at Copenhagen University Hospital, Hvidovre. It serves as a supplement to a quantitative investigation of an adapted MBSR course, prenatal MBSR, targeting pregnant women with psychosocial vulnerabilities. Interviews were conducted with five first-time mothers approximately one year postnatally to explore their experiences integrating elements from prenatal MBSR into their transition to motherhood. A reflexive thematic analysis was carried out. Four themes were generated to understand whether and how pregnant women with psychosocial vulnerabilities integrated elements from prenatal MBSR into the transition to motherhood and in relation to their child: (1) Awareness of the present moment; (2) Acting with consciousness towards oneself and one’s child; (3) Acceptance of oneself and one’s child, and (4) When it is difficult to integrate elements from prenatal MBSR. The findings suggested that prenatal MBSR may facilitate mother-child bonding and support the transition into motherhood for women with psychosocial vulnerabilities. Furthermore, the course may assist mothers in coping with challenges associated with this transition. Integrating MBSR practices into prenatal care may serve as a preventive intervention against maladaptive maternal adjustment and foster improved mother-infant relationships among populations with psychosocial vulnerabilities.

## Background

Motherhood is considered one of the most intense transitions in life, requiring preparation and adaptation [1,2]. This period is characterised by many changes [3] in which the women, among others, must develop a new identity as a mother and form a relationship with their child [4].Pregnancy and the transition to motherhood are often associated with happiness and meaning [5], but many women also experience significant stress and mental health problems during this period, due to emotional and physiological changes [6]. Mental disorders are a commonly encountered pregnancy complication, and women with a preconception history of mental disorders or psychosocial vulnerabilities are at increased risk [7]. The term *psychosocial* encompasses both psychological and social factors that can impact mental health [8]. In the *Good Start to Family Life* research program [9], which this study is part of, the term is used to describe a range of vulnerabilities that may pose a risk to maternal health or the health of the unborn child [10]. These vulnerabilities include, but are not limited to, a history of childhood abuse, low social support, intimate partner violence, previous trauma, current mood and anxiety disorders, or difficulty adjusting to pregnancy [10].

Untreated mental health disorders during pregnancy may be associated with adverse foetal development [11], including reduced foetal growth [12,13], preterm births [14,15], and harmful effects on infants’ development in regard to, e.g., cognitive, behavioural, and emotional issues [11,16]. Furthermore, mental health disorders can persist and/or elevate the risk for postnatal depression [17,18], which in turn can affect the mother-child bonding [19], making prevention essential for both the child and mother. There are thus strong arguments for the importance of strengthening prenatal mental health in pregnant women. Consequently, it is relevant to develop a deeper understanding of prenatal mental health interventions particularly for pregnant women with psychosocial vulnerabilities, which has informed this study.

Mindfulness-Based Stress Reduction (MBSR) is an evidence-based, non-pharmacological intervention designed to reduce stress and improve mental health [20]. MBSR is group-based and experiential, aimed at cultivating mindfulness and compassion. Mindfulness is defined by Jon Kabat-Zinn as “*the awareness that emerges through paying attention on purpose, in the present moment, and nonjudgmentally to the unfolding of experience moment by moment”* [21, p. 145], which is in the service of self-understanding, wisdom, and compassion [22]. Mindfulness-based interventions (MBIs) such as MBSR are acceptable for pregnant women with mental health difficulties [23] and address transdiagnostic mechanisms including decentering, self-compassion, meta-awareness, present-centred awareness, and nonreactivity [24,25]. Hence, MBSR could be an appropriate intervention to support pregnant women with psychosocial vulnerabilities. Therefore, an adapted version of MBSR was explored in the current study (see [9] for further details).

Studies have found that participation in prenatal MBIs is associated with increased mindfulness [26,27]. Moreover, a study reported that increased mindfulness was associated with decreased symptoms of anxiety and depression, as well as a self-reported better quality of the mother-infant bond 3–6 months postpartum [28]. This suggests that a higher degree of mindfulness is associated with a better mother-infant bond, which may be cultivated by participating in MBSR. However, there has been limited research conducted about how MBSR affects mother-infant bonding [29,30].

Generally, there is a growing body of evidence demonstrating that MBIs, including MBSR, are associated with reductions in symptoms of stress, anxiety, and depression in both a clinical and non-clinical context [31–33]. Similarly, there is growing interest in examining the effects of MBIs on anxiety, stress, and depression during pregnancy [27,34,35]. A systematic review and meta-analysis examined how MBIs affected stress, anxiety, and depression among perinatal women with and without current mental health issues [27]. Results showed that MBIs were effective in reducing depression, anxiety, and stress, and in increasing mindfulness among perinatal women with current mental health issues compared with a control group [27]. However, no significant differences were found between perinatal women without current mental health issues, who had participated in MBIs and those in the control group [27]. Another systematic review and meta-analysis examined the effect of MBIs on clinical and subthreshold perinatal depression and anxiety [34]. Significant and lasting effects were shown on symptoms of perinatal depression in both groups when compared with active and non-active control groups [34]. Results showed preliminary significant findings on anxiety, although more high-quality research is needed to confirm these effects [34]. An additional meta-analysis evaluated the efficacy of prenatal MBIs on postnatal symptoms of depression three months post-childbirth [35]. This meta-analysis differs from the above-mentioned studies in that both Chinese and English language studies were included. Findings showed significant effects on postnatal depression symptoms in women without a history of mental disorders, whereas no clear effects were identified among women with a history of depression or symptoms of anxiety and depression during pregnancy [35]. In sum, it can be argued that there are robust findings regarding the overall effect of MBIs on depressive symptoms among perinatal women. However, use of active control groups in research on MBI´s for perinatal mental health is limited and there is thus a need for more research applying stronger designs. Moreover, research on different subgroups such as women with/without current mental health issues and women with/without a history of mental health issues during pregnancy is warranted. The evidence supporting an effect on perinatal anxiety and stress is less robust due to a scarcity of randomized controlled trials which highlights the need for more research on perinatal anxiety and stress.

There is limited qualitative research on prenatal MBIs, and, to our knowledge, no qualitative studies exist on MBSR for pregnant women with psychosocial vulnerabilities. The available qualitative studies on prenatal MBIs differ from prenatal MBSR, for example, by including both parents [36]. However, one qualitative study examined the experience of prenatal Mindfulness-Based Cognitive Therapy (MBCT) among women with a history of anxiety and/or depression in Australia [37]. Participants interviewed postnatal valued the social support established during the intervention, reported improved present-moment awareness, and a greater acceptance of their experiences [37]. Unlike MBSR, which does not target a specific group or mental disorder, MBCT focuses on depression [38]. Furthermore, another qualitative study [39] investigated Prenatal Mindfulness Relationship-Based Program in a non-clinical population. The program is an 8-week group-based program delivered prenatally and with one follow-up session (10–12 weeks postnatal) informed by three key MBIs [20,36,38]. Results indicated that the program helped participants cope with postpartum challenges by developing an accepting attitude, present-moment awareness, and by being able to stop, breathe and notice thoughts, emotions and bodily sensations [39]. While these findings are promising, there remains a need to explore the experience of prenatal MBSR among pregnant women with psychosocial vulnerabilities.

Given the limited qualitative research on MBIs for pregnant women with psychosocial vulnerabilities, this study aimed to explore whether, and, if so, how participants in prenatal MBSR experienced being able to integrate elements from a course in prenatal MBSR into the transition to motherhood, and in relation to their child. The present qualitative interview study is part of a research program investigating prenatal MBSR for pregnant women with psychosocial vulnerabilities [9,10].

## Materials and methods

### Ethics statement

According to Danish legislation, formal ethical approval is not required in interview studies from the National Committee on Health Research Ethics [40]. However, this study adheres to the Declaration of Helsinki [41] and complies with the General Data Protection Regulation (GDPR; Regulation EU 2016/679). Participants provided written informed consent prior to the interviews, permitting the use of the material anonymously for a master thesis, and research purposes. An informed consent form, and participant information sheet about the study, was sent via email prior to the interviews. Given that the participants constitute a small, and highly selected group, significant measures were implemented to ensure their anonymity. This included the use of pseudonyms, aggregation of demographic information (see Table 1), and the exclusion of personally identifiable details in the results section, such as specific contexts, or references to recognisable individuals, locations, or events.

**Table 1:** Demographics characteristics of participants (N = 5).

	N	%
Age		
29-31	2	40
32-34	1	20
35-37	2	40
Cohabiting partner		
Yes	5	100
No	0	0
Occupation status		
Employed	3	60
Unemployed	1	20
Sick leave	1	20
Highest level of education		
Vocational college	1	20
Bachelor’s degree	3	60
Master’s degree	1	20
Reasons for referral		
Anxiety	1	20
Depression	2	40
Comorbid anxiety & depression	2	40
Infant gender		
Girl	2	40
Boy	3	60
Age of infant		
8-10 months	1	20
11-13 months	3	60
14-16 months	1	20

### Design and setting

The study was a qualitative interview study, consisting of five individual, semi-structured, online interviews with pregnant women, who had psychosocial vulnerabilities and had participated in prenatal MBSR [10]. Data collection and analysis followed an inductive methodological framework. Simultaneously, we adopted a flexible approach to the design of our qualitative study, utilising an iterative design [42]. Consistent with our iterative design, we revised the interview guide after observing that one question (“When did you participate in the mindfulness program?”) was difficult to answer for the participants and disrupted the interview flow. As this was not essential for the interview content, the question was removed. Furthermore, the thematic reflexive analysis was an iterative process in which the themes were continuously refined until they represented a comprehensive, consistent, and coherent understanding of the data, aligning with the approach described by Braun and Clarke [43].

The study was conducted in collaboration with the Danish Centre for Mindfulness, Aarhus University, and an obstetric ambulatory clinic at Copenhagen University Hospital, Hvidovre. The obstetric ambulatory clinic receives an average of 750 referrals per year and is one of five such clinics in Denmark that offer antenatal care to pregnant women with psychosocial vulnerabilities [10]. Pregnant women and their partners can be referred to the clinic due to a preconception history of one or more mental disorders, regardless of the current stage of illness, as well as complicating psychosocial vulnerabilities. Over 60% of the referrals to the clinic include a history of one or more mental disorders. The Danish Health Authority has outlined that the purpose of antenatal care is to mitigate the risk of obstetric complications and to promote a healthy pregnancy, which encompasses fostering maternal antenatal attachment. The obstetric ambulatory clinic employs a multidisciplinary approach, incorporating midwives, physicians, social workers, and psychologists. In Denmark, access to most healthcare services, including hospitals and general practitioners, is free and equitable. General practitioners serve as the primary gateway to all healthcare services. Pregnant women with psychosocial vulnerabilities are offered MBSR, adapted for pregnancy in a collaboration between the Danish Centre for Mindfulness and the obstetric ambulatory clinic, as a supplement to the usual care [10]. This course is called prenatal MBSR and is part of a larger initiative aimed at this target group [9]. The prenatal MBSR course was co-facilitated by two instructors: a certified MBSR teacher (second author, Lone Fjorback) and one midwife. A total of three midwives from the obstetric ambulatory clinic, all in training to become MBSR instructors, served as co-teachers across the courses. The research team consisted of people with different levels and areas of expertise. The first authors of this paper, Luna Berg Pedersen and Nanna Bjerg Ramsdal, were first master’s students in psychology and then newly educated psychologists at the time of writing, with prior experience with the course and training as MBSR instructors, but with limited experience in conducting qualitative research. Second author Lone Overby Fjorback is a specialist in psychiatry, researcher, and Associate Professor with many years of experience in MBSR. Third author Sophie Lykkegaard Ravn is a psychologist, researcher, and Associate Professor with experience in conducting qualitative studies, in particular using reflexive thematic analysis. Fourth author Michelle Kolls is a midwife and head of the obstetric ambulatory clinic at Copenhagen University Hospital, Hvidovre, with many years of experience working with pregnant women with psychosocial vulnerabilities. Last author Sine Skovbjerg is an Associate Professor with many years of experience with MBSR and research. The study adhered to the Consolidated Criteria for Reporting Qualitative Research (COREQ) [44].

### Study participants and data collection

Study participants were recruited using purposive sampling (by last author, Sine Skovbjerg) [45], whereby potential participants were contacted via email. Our inclusion and exclusion criteria align with those of Skovbjerg et al. [10]. In brief, inclusion criteria were: 1) estimated due date no sooner than three months from start of the intervention; 2) >18 years of age; 3) ability to speak and write Danish; and 4) provision of written informed consent to study participation. Exclusion criteria were: 1) an active substance dependence, 2) psychotic disorders (e.g., schizophrenia or bipolar disorder); 3) suicidality; and 4) being unavailable for two or more of the scheduled prenatal MBSR classes [10]. Participants with active substance dependence, psychotic disorders, or suicidality were excluded for ethical reasons, as they may require specialized care and could be adversely affected by MBSR [46,47]. Moreover, participation in the current study required completion of the prenatal MBSR course, i.e., attending at least five classes, and being a first-time mother, as the transition to motherhood is uniquely tied to the experience of having a first child [48]. Participants should also have a child around one year of age, as the purpose was to examine the significance of mindfulness when establishing the relationship between mother and child during the first year. A total of 16 participants were contacted. The recruitment period spanned from January 30th 2024 to March 13th. 2024. Of these, five participants agreed to participate in an interview (see Table 1 for demographic characteristics). Reasons for declining to participate were lack of time and resources. Interviews were conducted online via Zoom, and were audio recorded. Each participant was situated in their respective homes during the interview, which was conducted in March 2024 by the first authors (Luna Berg Pedersen & Nanna Bjerg Ramsdal). Each author functioned as the primary interviewer for two and three interviews, respectively, while the other author supplemented the interviews with follow-up questions and field notes during the interview. Both authors wrote down their impressions after the interview. The first authors had no prior acquaintance with the participants, whereas the second and last authors were familiar with the participants. An interview guide was constructed in collaboration between the first authors, second author, and last author, which guided the interviews. The interview guide first addressed participants’ understanding of mindfulness (e.g., “What do you understand by mindfulness?”). It then focused on their use of elements from prenatal MBSR in the transition to motherhood (e.g., “Is there anything from the mindfulness course that you use in becoming a mother?”). Finally, it examined how they applied prenatal MBSR in interactions with their child (e.g., “Have you used anything from the mindfulness course with your child?”). The interviewer asked follow-up questions and adjusted the order of questions based on participants’ responses. The interview guide is included as supporting information.. Each interview lasted one hour with a variation of +/– five minutes.

### Data analysis

The interviews and the analytic process were carried out in Danish. The audio records were first transcribed verbatim with the automatic speech recognition system Whisper, which is superior compared to similar programs [49]. It complies with General Data Protection Regulation (GDPR) and can thus be used for handling sensitive data [50]. Errors can occur when using automatic speech recognition systems [49], leading the first authors to listen through the audio-files, and review the transcriptions. Citations were translated into English for publication purposes by the first authors, making minor adjustments in the sentence structures for better comprehension in English. A reflexive thematic analysis, informed by Braun and Clarke [43,51] was conducted by the first authors. The reflexive aspect of thematic analysis highlights the researcher’s subjectivity in interpreting data, as the researcher’s preconceptions always influence their understanding of data [51]. Data was coded openly and inductively based on the material and not using a predetermined coding or theoretical framework. Of note, the first authors had in-depth prior knowledge about the adapted prenatal MBSR course, which will have impacted this open coding process. The initial phase of the analysis consisted of an overall familiarization with data. Afterwards, data was collaboratively coded by first authors, one transcript at a time. During this process, they discussed the coding for the given transcript, and developed a coding tree, which was iteratively expanded and refined as the analysis advanced. This coding process consisted of both semantic and latent codes [43,51]. Next, first authors identified patterns in the coded data, grouping the codes into preliminary multifaceted themes, with the help of the coding tree [51]. First authors then defined and refined themes by identifying the essence of what each theme was about, and selected quotes from the data were highlighted. While codes focused on more specific facets pertinent, themes were required to capture multiple facets relevant to answering the research question [51]. Throughout the data analysis process, a reflexive and organic approach was employed by which both codes and themes evolved dynamically [52]. The analysis was first conducted as part of the first authors’ master thesis, and later revised based on feedback provided by third author (Sophie Lykkegaard Ravn) and last author (Sine Skovbjerg), leading to adjustments, such as renaming themes to ensure that they captured their intended meaning. The subsequent section presents a narrative synthesis of the codes and themes, with supporting examples from data.

## Results

Four interrelated themes were developed, which contributed to an understanding of how participants in prenatal MBSR experienced being able to integrate elements from the course in the transition to motherhood, and in relation to their child: (1) *Awareness of the present moment*, (2) *Acting with consciousness towards oneself and one’s child*, (3) *Acceptance of oneself and one’s child*, and (4) *When it is difficult to integrate elements from prenatal MBSR.*

### Awareness of the present moment

The first theme, *Awareness of the present moment,* concerned being conscious of bodily sensations, the breath, thoughts, and emotions.

### Awareness of bodily sensations

All participants described being more aware of their bodily sensations after prenatal MBSR. They pointed to an experience of being better able to pause in situations, in which they noticed bodily changes. For example, Woman D stated:

“*I have gotten to know my body better, if that makes sense. I can better pinpoint ‘Okay, this is not normal. This is not how I feel when I am doing good. This is not how I feel when I am in balance.’ Then I think, ‘Okay, try to take a step back mentally.’*”

Similarly, all participants expressed that they were better able to notice bodily changes, listen to their body’s signals, and check in with themselves after prenatal MBSR. In doing this, participants provided examples of how they seemed to integrate elements from prenatal MBSR by using specific techniques from the course, such as checking in with themselves during their everyday life, as exemplified by Woman B:

“*The concrete thing I’ve taken with me from this course has been mini-meditations, or mini-body scans, to just check in with myself. Just to get some centred calm. To take those pauses during the day.*”.

In summary, the above indicates that the participants integrated elements from prenatal MBSR by having increased awareness of their bodies.

### Awareness of the breath

Four of the participants described becoming more aware of their breathing after prenatal MBSR, which can be related to the code *Awareness of bodily sensations*, as breathing can be seen as a sensation in the body. However, *Awareness of the breath* was very prominent in the participants’ descriptions, which is why it appears as an independent code. For example, Woman E described:

“*I really use breathing a lot, because as soon as the flow of thoughts starts, and if it begins to run a bit too fast, I think that the breathing often follows suit. It becomes very shallow in situations, in which one feels worried, perhaps panics, and a bit stressed. By somehow stopping, and taking some deep breaths, one can calm the nervous system.*”

Woman E described that she noticed the pace of her breathing tended to increase in line with the flow of thoughts when feeling worried, stressed, or in panic. This was also evident in the remaining three participants’ descriptions. All of them expressed anchoring their attention to the breath as a tool to regulate their thoughts. This was illustrated by Woman D:

“*But to feel ‘Now we’re breathing all the way down into the lungs’, and to feel them expand… and all that, because then we focus there, instead of all the mind chatter in our heads. And that allows you to say, ‘Now we’ve just taken a breath.’ Then you can tackle one thought at a time instead of having 40 thoughts at once*.”

Here, Woman D emphasized that breathing helped her shift focus from her thoughts to the physical sensations of her breathing, whereby she was able to focus on one thought at a time. As such, this may serve as an example of how attention to breathing can make thoughts more manageable. Taken together, these parts of data illustrate that participants integrated elements from prenatal MBSR by directing their attention to their breathing.

### Awareness of thoughts

All participants pointed to an experience of becoming more aware of their thoughts after participating in prenatal MBSR. This was particularly evident when they had a lot on their mind. For example, Woman B expressed:

“*It* [prenatal MBSR] *opened my eyes to be like, ‘God, I am in so many processes all the time. I’m sitting here, listening to a friend, but I’m also in the middle of... and I need to send that email... and I also need to remember... and oh no... and worries...’. I don’t do that anymore, and when I can feel it’s starting to show up, I try to shut it down. Either by finding awareness in my body, or it can be like, ‘Oh, now there’s a lot of mind chatter. I’m trying not to do anything about the thoughts. I just try to send them into the universe. I don’t need to use them right now.’*”

The statement above illustrates that Woman B, following prenatal MBSR, experienced becoming aware of spontaneous thoughts and worries about future tasks, as they occurred, allowing her to assess whether the thoughts were useful in the given moment. All participants described being aware of their thoughts as helpful in distancing themselves from them, e.g., by bringing attention to the body, or by acknowledging the thoughts and letting them pass. Several participants also mentioned that following prenatal MBSR, they had become aware of their thoughts while interacting with their child. Woman C described:

“*You sometimes find yourself in difficult situations with a very unhappy child. Especially in the beginning, when you might be afraid that something is wrong all the time (...). So, I used mindfulness a lot to breathe when I had those thoughts. So just focus on taking a step back, and breathing. And also, ‘Just be present in this. Be here and now. Right now, things are actually going really well, and it’s calm.’ I really think it has helped me not to get worked up.*”

All participants provided descriptions similar to the above, explaining that they stepped back when worries emerged, e.g., by shifting their attention to the breath and the present moment. This was found to be helpful in relation to their child, as the participants remained calm in challenging situations. Based on the above, it can be argued that participants experienced being able to integrate elements from prenatal MBSR into the transition to motherhood and in relation to their child, by being aware of thoughts.

### Awareness of emotions

All participants described increased awareness of their emotions following prenatal MBSR. For example, Woman B explained:

“*For me, it’s about getting in touch with myself, and notice how I feel. Being aware of how I am doing, what I am feeling, and where I am in life as well. So I think mindfulness for me could actually be replaced with the word awareness. It’s about being present. (...) And I am now aware that I was rarely present, aware of how I was doing, and conscious about what I did in my daily life. I have much more eye contact with people and listen much more now. I mean, I am more present in the moment.*”

As such, Woman B expressed that she was more attuned to her emotions in the present moment following prenatal MBSR. Similar descriptions were expressed by several other participants. This statement from Woman B not only illustrated the increased awareness of emotions, but also tapped into a more global sense of being present in the moment. Further, the statement also underlined that awareness of the present moment, including emotions, can influence the way one interacts relationally, which may be the case in relation to their children, but also to other people in their lives. In summary, the above suggests that the participants had become more aware of their emotions after prenatal MBSR, and that they may have integrated it in relation to their child.

### Acting with consciousness towards oneself and one’s child

The second theme, *Acting with consciousness towards oneself and one’s child,* captured the participants’ experience of being able to prioritise themselves, and letting go of what they think others think of them. Furthermore, the theme encompasses the participants’ experience of being able to regulate themselves in order to regulate their child, as well as being available for and present with their child.

### Prioritising oneself

All participants described that they experienced being better at prioritising themselves following prenatal MBSR. They expressed that they were more aware of their own needs and acted upon them. For example, Woman A described:

“*I have become better at saying, ‘Now I need a nap’, or ‘Now I need a bath’, or just to have some time for myself, and prioritise myself. Instead of thinking, ‘Now I need to be a mom’, or ‘Now I need to be a good girlfriend, a good sister, a good daughter, or whatever it might be... friend’. It’s also okay to be myself.*”

Here, Woman A described that she had become better at expressing her needs and prioritising herself rather than focusing on what she could do for others following prenatal MBSR. Woman A’s description can be seen as a conscious action, as she acts upon her own needs (e.g., taking a nap). Additionally, Woman D pointed out:

“*When the world falls apart, I can resort to saying, ‘I need to go by myself, and breathe and just focus on myself for a moment’. And then I can come back and be participative, present, a good mother, and at the same time be a good wife. Just be present in my surroundings and be able to look myself in the eyes.*”

Thus, Woman D described that she sensed her needs, voiced them, and acted upon them in stressful situations. It could be interpreted as a way of prioritising, and regulating herself, as she returned and acted more consciously in tune with her own values rather than acting automatically. This made her able to relate to others (e.g., her child) in a more desirable way. The other participants similarly described becoming aware of their own needs, and prioritising acting upon them. As such, it can be argued that all participants integrated elements from prenatal MBSR in the transition to motherhood, and in relation to their child.

### Letting go of what you think others think of you

Four out of the five participants provided descriptions that illustrated an improved ability to let go of concerns about others’ thoughts and perceptions of them following prenatal MBSR. Woman B described:

“*The difference from before to now is ‘What is the most important thing in life? It’s me and my family. What can I do something about, and what are the circumstances?’ I can’t waste energy thinking about what people think of me, because I can’t change it anyway, and it doesn’t have any effect on me or my child’s life, so ‘Who cares?’*”

Following prenatal MBSR Woman B experienced an increased awareness of her priorities in life, which she identified as herself and her family. It could be interpreted as a conscious action on a cognitive level when Woman B let go of her thoughts about others’ opinions of her, as she could not change them, and it did not affect her or her family. This was in line with the other participants’ descriptions. Woman C further pointed out that she experienced others frequently judging her approach to motherhood out loud. She stated:

“*There is a lot of comparison when you become a mother, and there is also a lot of mom-shaming. There are many ‘mom-cops’, right? So, everything you do can be criticized. (...) We have received many comments like ‘Oh, are you giving that to her?’ or ‘Oh, she should be sleeping now’ or things like that. It can really make me feel like ‘Is there something wrong with me? Am I such a bad and stupid mother?’ and all sorts of things. And then I use it* [mindfulness] *as a kind reminder to reconnect with myself and focus on positive thoughts instead of all those negative catastrophic thoughts.*”

Thereby she described consciously bringing awareness to positive thoughts instead of the negative ones when other people comment on her motherhood. As comparisons and comments from others may be evident towards mothers it can be helpful to have the capacity to let go of negative thoughts of oneself. Overall, it can be argued that the participants integrated elements from prenatal MBSR by consciously letting go of thoughts about other people’s opinions of them.

### Regulating oneself in order to regulate one’s child

All participants described that following prenatal MBSR, they experienced improved emotional regulation, enabling them to make more conscious behavioural choices concerning their child. This was apparent in Woman B’s statement:

“*We have a very mild boy, but when he gets upset, he’s almost inconsolable. And the crying of my child goes straight to my nervous system sending me directly into fight mode* (...)*. That’s when I’m particularly aware that I need to regulate my nervous system* (...)*. So, in this situation, we are good at either taking turns with our son, allowing one of us to step out and regulate, or simply sitting with our son, breathing, and bringing awareness back to the body again. And being like, ‘There’s no danger here. Everything is okay’, so we find calm, and I believe that helps our son a lot as well.*”

This statement exemplified Woman B’s ability to identify when she entered ‘fight mode’. Prenatal MBSR consists of psychoeducation about the behavioural responses, fight, freeze, and flight that can occur automatically in stressful situations. Woman B used this terminology to recognise the physical reactions she experienced, after which she regulated her nervous system. This may indicate that knowledge of stress responses may serve as a cue to regulate oneself, with the purpose of regulating the child, whereby it can be seen as a conscious action. Furthermore, Woman C attributed prenatal MBSR with helping her stay calm in challenging situations, which made her better able to regulate her child. She said:

“*I think it has helped me avoid getting worked up (...). It has helped me (...) to calm myself down first so that it can then calm my child down. I did that a lot in the beginning, and it wouldn’t have come naturally to me if I hadn’t gone through this mindfulness course while pregnant, when I was really struggling.*”

Woman C reflected on how prenatal MBSR had affected her ability to handle stressful situations as a mother. She highlighted the importance of calming herself first, in order to calm her child, which would not have come naturally had she not participated in prenatal MBSR. This suggests that mindfulness training can have a positive impact on the ability to self-regulate in challenging situations, whereby one avoids reacting negatively towards their child. Comparable descriptions appeared in the remaining interviews, suggesting that the participants incorporated elements from prenatal MBSR by consciously regulating themselves to effectively manage their child’s behaviour and emotions.

### Being available for and present with one’s child

Several participants articulated an awareness of being available and present during interactions with their children, which they attributed to prenatal MBSR, illustrated in the following statement:

Interviewer: “*Is there anything from this course that you have brought with you into this whole process of becoming a mother?*”

Woman A: “*Yes,* (...) *being in the moment, even when we’re sitting and playing.... or when my child wants to play on the floor... sometimes I can be a bit bad at... when my child doesn’t really want to interact, and my child’s just sort of wandering around by itself... My child’s good at that, fortunately... but sometimes you can end up taking out your phone, right? But we try to be aware that we shouldn’t be on the phone, so that when my child actually comes to us, we’re available and present.*”

As depicted above, Woman A consciously resisted the impulse to use her phone, in order to be available and present when her child sought her out. This may indicate that prenatal MBSR can facilitate becoming aware of the present moment, which can be applied to parent-child interactions. Woman B further pointed out that she was aware of moments when she was less present with her child:

“*And I can’t quite put my finger on what it is but I’ve completely changed the way I act and perceive the world. And I honestly believe that me and my child’s relationship would have been entirely different if I hadn’t been on that course, because I now notice when I feel stressed and pressured. For example, on days when I am under a lot of pressure, I’m not present with my child. I’m right next to it, and I play, but I’m not present. It could have been that way, I was a mother in relation to my child if I hadn’t done that course.*”

Woman B highlighted that she previously lacked awareness of feeling stressed or pressured, which resulted in her not being present. She credited prenatal MBSR as a significant factor, suggesting that prenatal MBSR had a positive impact on her ability to be present in parent-child interactions. Similar descriptions appeared in the other interviews, indicating that the participants experienced a capability to integrate elements from prenatal MBSR by being available for and present with their child.

### Acceptance of oneself and one’s child

The third theme, *Acceptance of oneself and one’s child,* captured the women’s acceptance of their inner experiences and their child’s development and emotions. Moreover, the theme concerned how the participants experienced that the group in prenatal MBSR played an important role in facilitating acceptance.

### Acceptance of inner experiences

All participants expressed that they had become more accepting of their inner experiences, following prenatal MBSR. Inner experiences refer to thoughts, emotions, and bodily sensations. All participants experienced increased acceptance of their thoughts and emotions while four participants also described greater tolerance of bodily sensations following prenatal MBSR. Woman E stated:

“*To tune into myself and allow thoughts and feelings to unfold. That wasn’t the biggest focus in my own upbringing, and I really want to give that to my child.*”

This statement indicates that Woman E accepted her thoughts and feelings whereby she broke with her parents’ patterns. This suggests that prenatal MBSR may facilitate an acceptance of thoughts and feelings, which subsequently may be passed on to their children. Furthermore, Woman B described a more accepting approach to her life following prenatal MBSR:

“*So, I’ve taken away the lesson that it’s okay not to be okay. (...) And that’s probably one of the newest things for me, this accepting, loving attention, because I’ve always been focused on thinking that I should feel better, and that I can’t be sad, so what can I do? (...) What I carry with me is a whole new approach to life. Accepting what is. Giving it space.*”

Thus, Woman B highlighted a completely new approach to her life, one in which she was better capable of accepting challenging emotions (e.g., sadness). The remaining participants similarly described an enhanced capacity to accept challenging emotions following prenatal MBSR. Additionally, Woman E described that body scans allowed her to approach her bodily sensations in a more accepting and observing way:

“*It’s about getting really close in a way that’s more about observing and less about fault-finding, if that makes sense. (...). Get close, but in a gentle way, and without assuming that it means something or needs to be fixed or resolved*”

The above statement can illustrate that woman E approached her bodily sensations in a more accepting way rather than a “fault-finding” way. The above statements point to an integration of elements from prenatal MBSR as the participants described a more observing, kind and accepting approach to their thoughts, feelings and bodily sensations in the present moment.

### Acceptance of one’s child

Four of the participants described being better able to accept their child following prenatal MBSR, which they found especially helpful in stressful situations. For example, Woman D pointed out:

“*Getting different tools* (...) *has made a huge difference for me in being able to hold space for my child when it’s angry and frustrated, and to hold space for myself when I am angry and frustrated at the same time as my child is angry and frustrated.*”

This can illustrate that Woman D experienced being able to accept her child’s anger and frustration, which she ascribed to tools from prenatal MBSR. It can be argued that the participants integrated elements from the course by accepting their child’s emotions as the descriptions of the remaining three participants resembled that of Woman D. Woman C further described:

“*You compare their development with other children’s. The other children in the mothers’ group can crawl and stand up. They’re almost walking. And then I think all sorts of things like ‘Is it because I’ve done something wrong?’* (...) *And it can quickly create a snowball effect, and then I try to zoom out of it, and say* (...) *‘It’s okay as it is right now.’ I can repeat that to myself, and then say to myself ‘But she’s so much better at socialising. So maybe that’s what she’s doing right now’. It is about being good at not getting caught up in the ‘There’s something wrong’-mindset.*”

Woman C expressed being able to “*zoom out of*” worries about her child’s development and shift perspective, so she focused on more positive aspects of her child’s development. She told herself that “*It’s okay as it is right now*”, which suggests an accepting approach to her daughter. This indicates that Woman C could decenter from her thoughts and thereby regulate herself, which may lead to a greater acceptance of her daughter’s development. In summary, the above examples illustrate how participants experienced integrating elements from prenatal MBSR by accepting their children’s emotions and development.

### The importance of the MBSR group to facilitate acceptance

Three participants highlighted the value of the group format in prenatal MBSR describing that the group played an important role in facilitating a more accepting approach towards themselves. Woman A pointed out:

“*And the girls I still talk to (...) have really meant a lot in accepting what is. ‘You’re not alone in this. There are others who feel the same way’, which makes it easier to be less judgemental towards yourself.*”

This statement implies that Woman A experienced a more accepting stance towards herself and was capable of embracing her situation, which she attributed to the other members of the course as she mirrored her experiences of becoming a mother in other members’ descriptions. At the same time, it can be seen as an expression of normalisation of her challenges, whereby acceptance of the challenges was facilitated. Similar descriptions were found in the remaining two interviews. This illustrates that the group in prenatal MBSR was experienced as an aid in promoting acceptance towards oneself in the transition to motherhood.

### When it is difficult to integrate elements from prenatal MBSR

The fourth and final theme, *When it is difficult to integrate elements from prenatal MBSR,* was related to situations and periods of stress and pressure in which participants found it challenging to remember, prioritise and integrate what they had learnt from prenatal MBSR.

### Periods and situations with stress and pressure

All five participants described situations or periods in which they found it difficult to integrate elements from prenatal MBSR. Woman E pointed out:

*It’s something you need to prioritise, and I need to have some degree of energy before I can engage in some of those thought patterns. And there have been times when we’ve felt under pressure at home, and we’ve been sick, and there have been other things going on, where I know it would help me if I just sat down for 10 minutes and did a body scan* (...) *but I haven’t been able to find the energy to do it.*”

Woman E highlighted that she found it difficult to prioritise using elements from prenatal MBSR (e.g., body scan) during stressful periods with little energy, which were similarly evident in other interviews. This suggests that prioritising formal mindfulness practices (e.g., body scan), may become particularly challenging when individuals experience low energy levels despite awareness of its benefits. Woman A further shared:

“*I think I forget to use it once I’ve gone too far off track. If I don’t remember to maintain it, which can be really difficult when your everyday life is just running with daycare and work. And remembering to pull the plug in time and say, ‘I need a break now’ (...). When I don’t remember to pull the plug in time, I forget everything I’ve learnt if I’m under pressure.*”

The quote highlights that Woman A struggled to integrate practices learnt in prenatal MBSR into the demands of a busy daily life. She pointed out that it’s necessary to “maintain it”. This can indicate the importance of actively remembering to practise mindfulness in order to integrate elements from prenatal MBSR, such as listening to needs and acting on them. Furthermore, some participants mentioned that it was difficult to integrate in specific situations. Woman C for example described:

“*When my child was very little, it had a lot of stomach issues, and we couldn’t really do anything about it (...). So, when I was there, and my child was just crying so much, and I almost wanted to sit down and cry myself (...) I couldn’t always use it. But I think I’ve used it afterwards, because then I can get caught up in thinking that I’m not good enough. If a situation doesn’t go well* (...) *I actually use it more retrospectively, by looking at myself lovingly, and trying to calm down those thoughts, not giving them attention.*”

Thus, the statement can illustrate that Woman C experienced difficulty integrating prenatal MBSR techniques during immediate, challenging situations with her child, yet she was able to implement them afterwards. This can suggest that it can be difficult to integrate elements in specific demanding situations, though it can be used retrospectively by having a loving approach towards oneself and not focusing on negative thoughts. Overall, the above suggests that participants found it difficult to integrate elements from prenatal MBSR in some situations and periods with stress and pressure. Despite this, the statements can be interpreted as suggesting that participants were still able to integrate principles from prenatal MBSR. For example, by speaking lovingly to themselves after the difficult situation, and having a certain level of awareness of their reaction patterns under stress and pressure, which they tried to address.

## Discussion

### Summary of findings

The present study aimed to investigate whether and, if so, how participants in prenatal MBSR experienced being able to integrate elements from the course into the transition to motherhood and in relation to their child. This was explored in a qualitative interview study, generating four themes. The first theme showed that participants of prenatal MBSR experienced being able to integrate elements from the course by anchoring their attention on present-moment experiences, including bodily sensations, the breath, thoughts, and emotions. Additionally, the second theme pointed to an integration of elements by acting consciously towards themselves and their child. This included being able to prioritise and act upon their needs, letting go of what they thought others thought of them, regulating their emotions with a purpose of regulating their child and remaining mentally available for their child. Furthermore, the participants’ descriptions in the third theme pointed to an integration of elements from the course regarding acceptance. Specifically, they described being able to accept their own inner experiences (e.g., thoughts, emotions, and bodily sensations) and their child’s emotions and developmental stage. The acceptance towards themselves and their child was facilitated by the prenatal MBSR group. However, the fourth theme highlighted that challenges in integrating elements of the course emerged during some periods of stress. Participants found it demanding to prioritise formal practice, although they expressed being aware of the benefits of practice in these periods. They furthermore described it as challenging to integrate in some difficult situations (e.g., with a consolable child) in which their statements could indicate a tendency to react automatically rather than consciously. Despite this, their statements reflect an ability to integrate it retrospectively by having a loving stance towards themselves and, in the moment, by bringing awareness to their inner experiences, thereby demonstrating an ability to incorporate elements from the course. Hence, the overall findings indicate that participants experienced being able to integrate elements from prenatal MBSR into the transition to motherhood and in relation to their child.

### Discussion and contextualisation of findings

The findings in the present study highlight the participants ability to bring awareness to the present moment, act consciously, and be accepting towards experiences, which can be seen as core aspects of mindfulness, cultivated in the prenatal MBSR course. The definition of mindfulness by Jon Kabat-Zinn [21] can be related to the aforementioned themes as participants described a non-judgemental awareness towards their experiences in the moment. Thus, participants demonstrate good adherence to the course approximately one year postnatally, even though their formal practice was limited. This can be seen in relation to the overall findings of quantitative research on prenatal MBIs which demonstrated reductions in postnatal depressive and anxiety symptoms. These findings suggest that such interventions may play a beneficial role in supporting the transition to motherhood and have a long-term effect [34,35].

There is an increasing interest in prenatal MBIs for pregnant women with psychosocial vulnerabilities (e.g., women with depression or anxiety) [27,34,35], yet few studies have investigated prenatal MBIs qualitatively. The overarching themes developed in the present study are in line with earlier qualitative research on prenatal MBIs [37,39]. Previous studies reported that participants, after engaging in the investigated prenatal MBI, experienced increased acceptance, enhanced present-moment awareness, and a greater ability to stop and notice bodily sensations, emotions, and thoughts, enabling them to consciously respond rather than react automatically to stimuli [37,39]. These findings align with the results of the first three themes in the present study. However, findings of the third theme in this study differ from the earlier studies by highlighting the importance of keeping contact with group members in the transition to motherhood. Furthermore, results of the current study pointed to periods and situations, in which the participants described it as challenging to apply elements from prenatal MBSR (e.g., formal practice or having a loving approach towards oneself).

As mentioned above, participants in prenatal MBSR described an ability to step back from their current thoughts and feelings, an awareness that thoughts and emotions will pass, and a non-judgemental stance towards thoughts and feelings, which is in line with previous qualitative findings [37,39]. This can point to the potential mechanism of decentering [24], which is defined as an ability to observe thoughts and emotions as temporary mental events as opposed to true reflections of the self or reality [53]. Furthermore, the findings of the present study suggest that self-compassion might be a potential mechanism of prenatal MBSR, which is in line with existing quantitative research [24]. According to Kristin Neff, self-compassion encompasses three components: 1) kindness towards oneself, 2) recognition of a common humanity, acknowledging that everyone makes mistakes, and 3) mindfulness, which involves awareness of emotions and thoughts [54]. This is seen as participants described a more accepting and loving stance towards themselves, an awareness of, e.g., thoughts and emotions as well as the group’s importance in acknowledging that everyone finds parenting difficult. Previous research found similar descriptions, which can be related to the three components of self-compassion [37,39]. However, the current study adds to this understanding by highlighting that the experience of common humanity can be sustained and even strengthened following the course, provided participants maintain contact with the group. These potential mechanisms suggest that future research should explore whether, and if so how, self-compassion and decentering are strengthened through prenatal MBSR, and their contribution to fostering more adaptive coping strategies during the transition into motherhood and in the parent-child relationship.

### Discussion of clinical perspectives

Based on the above, it can be argued that the participants were able to integrate what they had learnt during prenatal MBSR approximately one year postnatal, which points to a considerable clinical impact of the course for the participants. Since the participants consist of a clinical population, their mental health challenges can affect their child adversely [11,16,19]. This underscores the importance and relevance of prenatal MBSR and of intervening during this window of opportunity. The results of the current study highlight that it can be difficult to respond consciously in some challenging situations and maintain formal practice in periods with pressure and low energy. As the transition to motherhood is a period characterised by many emotional and physical changes, which can be considered stressful [6], there is a need for strengthening the participants’ practice and additional support in the early stages of motherhood. The maintenance of formal practice is important, as a study showed that it is associated with more life satisfaction, less stress and more mindfulness [55]. Moreover, attending mindfulness groups and courses [55] and an established meditation routine [55–57] are helpful in supporting a mindfulness practice. Despite the benefits of a meditation routine, it can be considered challenging to establish amidst the demands of caring for an infant. This can point to the relevance of focusing on more informal practice during everyday life (e.g., mini-meditations) in prenatal MBSR. Mini-meditations can make it easier to integrate mindfulness in everyday life as it can help the individual to step back from their automatic behaviour on stressful days, enabling an awareness of the present moment [58]. Based on the above, it can furthermore be relevant to create opportunities, whereby participants can attend mindfulness groups postnatally. In prenatal MBSR, an online booster session is offered three months postnatally, yet clinical experience with the course shows low attendance rates. Alternatively, it could be relevant to offer a weekly online drop-in session for previous participants of prenatal MBSR, as it is more likely that the participants are able to attend some of the sessions. Furthermore, the results of the current study showed that maintaining contact and sharing difficulties with group members could foster integration of elements from prenatal MBSR in the transition to motherhood. In light of this, it can be argued that the drop-in sessions can help previous course members to maintain contact with each other. Future studies should investigate how to support the maintenance of mindfulness practice following prenatal MBSR. In this regard, it could be relevant to explore how to facilitate maintenance of the group and the development of a meditation routine.

### Limitations

Although the study design does not allow for definitive conclusions regarding the potential long-term effects of prenatal MBSR, the findings may suggest that the course can help pregnant women with psychosocial vulnerabilities in the transition to motherhood and in relation to their child. However, important limitations should be highlighted for future research. In this regard, the representativeness of the sample can be a possible limitation. All participants were referred to prenatal MBSR due to a psychosocial vulnerability (e.g., anxiety and depression), whereby they may represent the target group. A potential limitation to consider in the study is selection bias. To address this, all women who meet our in- and exclusion criteria were contacted in the recruitment process. Although, an inclusion criteria in the current study was completion of the prenatal MBSR course (i.e., attending at least five classes), which might have limited perspectives of the participants who might have a less favourable view of prenatal MBSR. In this regard participants who are willing to engage in an interview may be more inclined to have a positive perception of the course, gained the most from the intervention and/or have more resources to engage in such additional activities compared with the participants who did not complete the course. Despite this, two-thirds of the women invited to participate in the current study declined, citing a lack of time and energy. This suggests that they may not have had the necessary resources to engage in the study although they completed the course. This is not an uncommon phenomenon when recruiting participants from less privileged circumstances for qualitative studies [59], but is nonetheless important to have in mind when interpreting the results. As such, the participants may not represent the full spectrum of women that receive prenatal MBSR. Due to this non-response sampling bias, experiences of adverse effects or no effects may be overlooked. In this context, it is important to consider that resource availability is not solely an individual matter, but often shaped by broader structural conditions such as logistics (e.g., transportation time to the Obstetric Ambulatory Clinic), socioeconomic status (e.g., work status) and barriers in the healthcare system (e.g., attitudes towards minority groups). These factors may have limited some women’s ability to complete the course or participate in the study.

Some limitations might also be present in executing qualitative interviews. In light of this, social desirability bias is a known limitation, whereby participants might say what they believe the interviewer wants to hear [60]. This could be the case in the current study as participants were recruited by last author, who was also involved in the prenatal MBSR. However, techniques to minimise this issue were employed, including briefing on the purpose of the present study and the procedure for ensuring confidentiality and anonymity, fostering a safe atmosphere, and validating the participants’ statements [60]. Furthermore, the interviewer explicitly inquired about situations in which participants encountered difficulties in applying techniques from prenatal MBSR. These inquiries were framed to encourage participants to share any adverse or challenging experiences they had with the course.

Due to the design of the current study, it cannot be determined with certainty whether the differences experienced by the participants following prenatal MBSR are directly attributable to the course, as many changes naturally occur during pregnancy [6]. Although this is the case, pregnancy is also seen as a window of opportunity to change maladaptive emotional and behavioural patterns, which can be fostered by interventions in this period. Since the findings of the current study can be related to quantitative research on prenatal MBI’s, for example showing decreased symptoms of anxiety and depression postnatal [34,35], it can be argued that participant experiences indeed can be attributable to the course.

An additional limitation of the study is the risk of subtle shifts in meaning during the translation of interview quotations from Danish to English. While care was taken to preserve the original tone and intent, some nuances may have been altered in the process. To mitigate this, the first authors collaborated closely during translation and engaged in ongoing discussions to ensure accuracy. Furthermore, the entire research team had access to both the Danish and English versions of the quotations and if the first authors encountered difficulties with the translations, the other authors were consulted during manuscript review to ensure that the original meaning was preserved.

## Conclusion

The purpose of the present study was to explore whether, and if so, how participants in prenatal MBSR experienced being able to integrate elements from the course into the transition to motherhood and in relation to their child. This study utilised semi-structured interviews with five first-time mothers, who had participated in prenatal MBSR for pregnant women with psychosocial vulnerabilities. Using a reflexive thematic analysis, four main themes and 12 related codes were developed. This study highlights several findings. Participants experienced being able to integrate elements from prenatal MBSR by being aware of the present moment, acting with awareness, and adopting an accepting stance toward themselves and their child. However, participants described some periods and situations (e.g., stress and low energy), in which they found it challenging to apply what they had learnt during the course. Despite the limitations and considerations of the study, the findings point to potential long-term benefits of prenatal MBSR in the transition to motherhood and in relation to their child. Taking the limited qualitative research in this area into consideration, we argue that the current study addresses a knowledge gap regarding prenatal MBIs for a pregnant population with psychosocial vulnerabilities, highlighting experienced long-term benefits in prenatal MBSR. However, the outlined limitations are important to consider. Future studies should focus on identifying methods to sustain these benefits over time.

## Supporting information

S1 File Interview guide. (PDF)

## References

[1] Biaggi A, Conroy S, Pawlby S, Pariante CM. Identifying the women at risk of antenatal anxiety and depression: A systematic review. J Affect Disord. 2016;191:62–77. doi: 10.1016/j.jad.2015.11.014 26650969 PMC4879174

[2] Richter N, Bondü R, Trommsdorff G. Linking transition to motherhood to parenting, children’s emotion regulation, and life satisfaction: A longitudinal study. J Fam Psychol. 2022;36(2):291–300. doi: 10.1037/fam0000868 34060894

[3] Miller LJ. Psychological, behavioral, and cognitive changes during pregnancy and the postpartum period. The Oxford handbook of perinatal psychology. New York, NY, US: Oxford University Press. 2016. p. 7–25.

[4] Pajulo M, Tolvanen M, Karlsson L, Halme-Chowdhury E, Öst C, Luyten P, et al. The prenatal parental reflective functioning questionnaire: exploring factor structure and construct validity of a new measure in the finn brain birth cohort pilot study. Infant Ment Health J. 2015;36(4):399–414. doi: 10.1002/imhj.21523 26096692

[5] Wenzel A. Introduction: The unique importance of perinatal psychology. The Oxford Handbook of Perinatal Psychology. Oxford University Press. 2015. p. 0.

[6] Trapani S, Caglioni M, Villa G, Manara DF, Caruso R. Mindfulness-based interventions during pregnancy and long-term effects on postpartum depression and maternal mental health: a systematic review and meta-analysis of randomized controlled trials. J Integr Complement Med. 2024;30(2):107–20. doi: 10.1089/jicm.2023.0114 37638799

[7] Howard LM, Molyneaux E, Dennis C-L, Rochat T, Stein A, Milgrom J. Non-psychotic mental disorders in the perinatal period. Lancet. 2014;384(9956):1775–88. doi: 10.1016/S0140-6736(14)61276-9 25455248

[8] Vizzotto ADB, de Oliveira AM, Elkis H, Cordeiro Q, Buchain PC. Psychosocial characteristics. In: Gellman MD, Turner JR, editors. New York, NY: Springer New York. 2013. p. 1578–80.

[9] Skovbjerg S, Birk D, Bruggisser S, Wolf ALA, Fjorback L. Mindfulness-based stress reduction adapted to pregnant women with psychosocial vulnerabilities-a protocol for a randomized feasibility study in a Danish hospital-based outpatient setting. Pilot Feasibility Stud. 2021;7(1):118. doi: 10.1186/s40814-021-00860-w 34082839 PMC8173971

[10] Skovbjerg S, Sumbundu A, Kolls M, Kjærbye-Thygesen A, Fjorback LO. The effect of an adapted Mindfulness-Based Stress Reduction program on mental health, maternal bonding and birth outcomes in psychosocially vulnerable pregnant women: a study protocol for a randomized controlled trial in a Danish hospital-based outpatient setting. BMC Complement Med Ther. 2023;23(1):364. doi: 10.1186/s12906-023-04194-3 37838672 PMC10576273

[11] Stein A, Pearson RM, Goodman SH, Rapa E, Rahman A, McCallum M, et al. Effects of perinatal mental disorders on the fetus and child. Lancet. 2014;384(9956):1800–19. doi: 10.1016/S0140-6736(14)61277-0 25455250

[12] Jarde A, Morais M, Kingston D, Giallo R, MacQueen GM, Giglia L, et al. Neonatal Outcomes in Women With Untreated Antenatal Depression Compared With Women Without Depression: A Systematic Review and Meta-analysis. JAMA Psychiatry. 2016;73(8):826–37. doi: 10.1001/jamapsychiatry.2016.0934 27276520

[13] Ding X-X, Wu Y-L, Xu S-J, Zhu R-P, Jia X-M, Zhang S-F, et al. Maternal anxiety during pregnancy and adverse birth outcomes: a systematic review and meta-analysis of prospective cohort studies. J Affect Disord. 2014;159:103–10. doi: 10.1016/j.jad.2014.02.027 24679397

[14] Grote NK, Bridge JA, Gavin AR, Melville JL, Iyengar S, Katon WJ. A meta-analysis of depression during pregnancy and the risk of preterm birth, low birth weight, and intrauterine growth restriction. Arch Gen Psychiatry. 2010;67(10):1012–24. doi: 10.1001/archgenpsychiatry.2010.111 20921117 PMC3025772

[15] Grigoriadis S, VonderPorten EH, Mamisashvili L, Tomlinson G, Dennis C-L, Koren G, et al. The impact of maternal depression during pregnancy on perinatal outcomes: a systematic review and meta-analysis. J Clin Psychiatry. 2013;74(4):e321-41. doi: 10.4088/JCP.12r07968 23656857

[16] Glasheen C, Richardson GA, Fabio A. A systematic review of the effects of postnatal maternal anxiety on children. Arch Womens Ment Health. 2010;13(1):61–74. doi: 10.1007/s00737-009-0109-y 19789953 PMC3100191

[17] Zhao X-H, Zhang Z-H. Risk factors for postpartum depression: An evidence-based systematic review of systematic reviews and meta-analyses. Asian J Psychiatr. 2020;53:102353. doi: 10.1016/j.ajp.2020.102353 32927309

[18] Beck CT. Predictors of postpartum depression: an update. Nurs Res. 2001;50(5):275–85. doi: 10.1097/00006199-200109000-00004 11570712

[19] Erickson N, Julian M, Muzik M. Perinatal depression, PTSD, and trauma: Impact on mother-infant attachment and interventions to mitigate the transmission of risk. Int Rev Psychiatry. 2019;31(3):245–63. doi: 10.1080/09540261.2018.1563529 30810410

[20] Kabat-Zinn J. Full catastrophe living: how to cope with stress, pain and illness using mindfulness meditation. Revised and updated edition ed. London: Piatkus. 2020.

[21] Kabat-Zinn J. Mindfulness-based interventions in context: Past, present, and future. Clinical Psychology: Science and Practice. 2003;10(2):144–56. doi: 10.1093/clipsy.bpg016

[22] Kabat-Zinn J. Meditation is not what you think. Mindfulness. 2021;12(3):784–7.

[23] Shi Z, MacBeth A. The Effectiveness of Mindfulness-Based Interventions on Maternal Perinatal Mental Health Outcomes: a Systematic Review. Mindfulness (N Y). 2017;8(4):823–47. doi: 10.1007/s12671-016-0673-y 28757900 PMC5506176

[24] Davis KM, Wojcik CM, Baillie AJ, Foley E, Goddard T, Lau MA, et al. Mechanisms of mindfulness: a longitudinal study of a mindfulness-based stress reduction program. Mindfulness. 2024;15(5):1188–207. doi: 10.1007/s12671-024-02359-w

[25] Wielgosz J, Goldberg SB, Kral TRA, Dunne JD, Davidson RJ. Mindfulness Meditation and Psychopathology. Annu Rev Clin Psychol. 2019;15:285–316. doi: 10.1146/annurev-clinpsy-021815-093423 30525995 PMC6597263

[26] Goodman RJ, Quaglia JT, Brown KW. Burning issues in dispositional mindfulness research. Handbook of mindfulness and self-regulation. New York, NY, US: Springer Science Business Media. 2015. p. 67–80.

[27] Yan H, Wu Y, Li H. Effect of mindfulness-based interventions on mental health of perinatal women with or without current mental health issues: A systematic review and meta-analysis of randomized controlled trials. J Affect Disord. 2022;305:102–14. doi: 10.1016/j.jad.2022.03.002 35257692

[28] McDonald HM, Sherman KA, Kasparian NA. How Mindful Awareness and Psychological Distress Influence Mother-Infant Bonding and Maternal Perceptions of Infant Temperament. Mindfulness. 2022;13(4):955–66. doi: 10.1007/s12671-022-01848-0

[29] Ahemaitijiang N, Fang H, Ren Y, Han ZR, Singh NN. A review of mindful parenting: Theory, measurement, correlates, and outcomes. Journal of Pacific Rim Psychology. 2021;15:18344909211037016.

[30] Shorey S, Ng ED. The efficacy of mindful parenting interventions: A systematic review and meta-analysis. Int J Nurs Stud. 2021;121:103996. doi: 10.1016/j.ijnurstu.2021.103996 34175531

[31] Galante J, Friedrich C, Collaboration of Mindfulness Trials (CoMinT) 3, Dalgleish T, Jones PB, White IR, et al. Individual participant data systematic review and meta-analysis of randomised controlled trials assessing adult mindfulness-based programmes for mental health promotion in non-clinical settings. Nat Ment Health. 2023;1(7):462–76. doi: 10.1038/s44220-023-00081-5 37867573 PMC7615230

[32] Goldberg SB, Riordan KM, Sun S, Davidson RJ. The empirical status of mindfulness-based interventions: a systematic review of 44 meta-analyses of randomized controlled trials. Perspect Psychol Sci. 2022;17(1):108–30. doi: 10.1177/1745691620968771 33593124 PMC8364929

[33] Kuyken W, Warren FC, Taylor RS, Whalley B, Crane C, Bondolfi G, et al. Efficacy of mindfulness-based cognitive therapy in prevention of depressive relapse: an individual patient data meta-analysis from randomized trials. JAMA Psychiatry. 2016;73(6):565–74. doi: 10.1001/jamapsychiatry.2016.0076 27119968 PMC6640038

[34] Leng LL, Yin XC, Ng SM. Mindfulness-based intervention for clinical and subthreshold perinatal depression and anxiety: A systematic review and meta-analysis of randomized controlled trial. Compr Psychiatry. 2023;122:152375. doi: 10.1016/j.comppsych.2023.152375 36841089

[35] Min W, Jiang C, Li Z, Wang Z. The effect of mindfulness-based interventions during pregnancy on postpartum mental health: A meta-analysis. J Affect Disord. 2023;331:452–60. doi: 10.1016/j.jad.2023.03.053 36963518

[36] Duncan LG, Bardacke N. Mindfulness-Based Childbirth and Parenting Education: Promoting Family Mindfulness During the Perinatal Period. J Child Fam Stud. 2010;19(2):190–202. doi: 10.1007/s10826-009-9313-7 20339571 PMC2837157

[37] Dunn C, Hanieh E, Roberts R, Powrie R. Mindful pregnancy and childbirth: effects of a mindfulness-based intervention on women’s psychological distress and well-being in the perinatal period. Arch Womens Ment Health. 2012;15(2):139–43. doi: 10.1007/s00737-012-0264-4 22382281

[38] Segal Z, Williams M, Teasdale J, Kabat-Zinn J. Mindfulness-Based Cognitive Therapy for Depression. 2 ed. New York: Guilford Publications. 2012.

[39] Sansone A, Stapleton P, Patching A. A qualitative investigation of a prenatal mindfulness relationship-based (PMRB) program to support maternal mental health and mother–baby relationship during pregnancy and post-partum. Mindfulness. 2024;15(7):1759–77. doi: 10.1007/s12671-024-02399-2

[40] Bekendtgørelse af lov om videnskabsetisk behandling af sundhedsvidenskabelige forskningsprojekter og sundhedsdatavidenskabelige forskningsprojekter: Anmeldelsespligt. Denmark. 2020. https://www.retsinformation.dk/eli/lta/2020/1338#P14

[41] World Medical Association. World Medical Association Declaration of Helsinki. Ethical principles for medical research involving human subjects. Bull World Health Organ. 2001;79(4):373–4. 11357217 PMC2566407

[42] Rubin H, Rubin I. Qualitative Interviewing: The Art of Hearing Data. 2nd ed. Thousand Oaks, California: SAGE Publications, Inc. 2005.

[43] Braun V, Clarke V. Using thematic analysis in psychology. Qualitative Res Psychol. 2006;3(2):77–101. doi: 10.1191/1478088706qp063oa

[44] Tong A, Sainsbury P, Craig J. Consolidated criteria for reporting qualitative research (COREQ): a 32-item checklist for interviews and focus groups. Int J Qual Health Care. 2007;19(6):349–57. doi: 10.1093/intqhc/mzm042 17872937

[45] Palinkas LA, Horwitz SM, Green CA, Wisdom JP, Duan N, Hoagwood K. Purposeful sampling for qualitative data collection and analysis in mixed method implementation research. Adm Policy Ment Health. 2015;42(5):533–44. doi: 10.1007/s10488-013-0528-y 24193818 PMC4012002

[46] Baer R, Crane C, Miller E, Kuyken W. Doing no harm in mindfulness-based programs: Conceptual issues and empirical findings. Clin Psychol Rev. 2019;71:101–14. doi: 10.1016/j.cpr.2019.01.001 30638824 PMC6575147

[47] Santorelli S, Maleo-Meyer F, Koerbel L, Kabat-Zinn J. Mindfulness-based stress reduction (MBSR) authorized curriculum guide. University of Massachusetts Medical School. 2017.

[48] Oliveira JM de, Alvarenga P, Paixão C, Sales PKC. Systematic Review of Interventions with Parents in the Transition to Parenthood. PTP. 2023;25(2). doi: 10.5935/1980-6906/eptpcp14839.en

[49] Wollin-Giering S, Hoffmann M, Höfting J, Ventzke C. Automatic transcription of English and German qualitative interviews. Forum, Qualitative Social Research. 2024;25(1):1–37.

[50] Center SU e. Secure Platform. https://docs.cloud.sdu.dk/intro/security.html

[51] Braun V, Clarke V. One size fits all? What counts as quality practice in (reflexive) thematic analysis?. Qualitative Res Psychol. 2020;18(3):328–52. doi: 10.1080/14780887.2020.1769238

[52] Braun V, Clarke V. Reflecting on reflexive thematic analysis. Qualitative Research in Sport, Exercise and Health. 2019;11(4):589–97. doi: 10.1080/2159676x.2019.1628806

[53] Fresco DM, Moore MT, van Dulmen MHM, Segal ZV, Ma SH, Teasdale JD, et al. Initial psychometric properties of the experiences questionnaire: validation of a self-report measure of decentering. Behavior Therapy. 2007;38(3):234–46. 17697849 10.1016/j.beth.2006.08.003

[54] Neff K. Self-Compassion: An Alternative Conceptualization of a Healthy Attitude Toward Oneself. Self and Identity. 2003;2(2):85–101. doi: 10.1080/15298860309032

[55] Beblo T, Haehnel K, Michalak J, Iffland B, Driessen M. Integrating mindfulness practice into everyday life after completing a course in mindfulness-based stress reduction. Nordic Psychol. 2024;76(4):506–18. doi: 10.1080/19012276.2024.2303432

[56] Yavuz Sercekman M. Exploring the sustained impact of the Mindfulness-Based Stress Reduction program: a thematic analysis. Front Psychol. 2024;15:1347336. doi: 10.3389/fpsyg.2024.1347336 39100567 PMC11294918

[57] Masheder J, Fjorback L, Parsons CE. “I am getting something out of this, so I am going to stick with it”: supporting participants’ home practice in Mindfulness-Based Programmes. BMC Psychol. 2020;8(1):91. doi: 10.1186/s40359-020-00453-x 32867834 PMC7457766

[58] Baer RA. Introduction to the core practices and exercises. Mindfulness-based treatment approaches: Clinician’s guide to evidence base and applications. 2nd ed. ed. San Diego, CA, US: Elsevier Academic Press. 2014. p. 3–25.

[59] Serrano-Gallardo P, Cassetti V, Boone ALD, Pisano-González MM. Recruiting Participants in Vulnerable Situations: A Qualitative Evaluation of the Recruitment Process in the EFFICHRONIC Study. Int J Environ Res Public Health. 2022;19(17):10765. doi: 10.3390/ijerph191710765 36078487 PMC9518307

[60] Bergen N, Labonté R. “Everything is perfect, and we have no problems”: detecting and limiting social desirability bias in qualitative research. Qual Health Res. 2020;30(5):783–92. doi: 10.1177/1049732319889354 31830860

