# Supplementary material for: “This course has made it easier for me to embrace myself and my child”: A qualitative study of an adapted mindfulness-based stress reduction course for pregnant women with psychosocial vulnerabilities
Source: PLOS Ment Health. 2025 Nov 7;2(11):e0000412. doi: 10.1371/journal.pmen.0000412 (PMC12798619; doi:10.1371/journal.pmen.0000412)
Supplement: S1 File — (PDF) [file pmen.0000412.s001.pdf]

S1: Interviewguide

**Briefing before the interview**		
Introduction of Interviewer and Observer	Who are we?	My name is Nanna/Luna. We spoke on the phone earlier. I am studying psychology in my 10th semester at Aarhus University. I am working on my thesis together with Nanna/Luna.<br><br>My name is Nanna/Luna. I am also in my 10th semester of psychology studies at Aarhus University.
Framework for interview	My role in the interview<br><br>Structure of the interview	I will be asking questions throughout the interview, and Luna/Nanna may ask follow-up questions towards the end.<br><br>The interview will first focus on your understanding of mindfulness. Then we will talk about becoming a mother, and whether you’ve experienced using elements from the mindfulness course for yourself and your child.<br><br>We’ll spend some time asking about concrete experiences you’ve had with mindfulness. It may feel a bit silly to spend that much time on it, but it's to get as close as possible to your actual experience.
Presentation of the project		**Thesis**: This project is conducted in collaboration with the Danish Center for Mindfulness and Amager/Hvidovre Family Outpatient Clinic. It will be used for our thesis, and also published as a research article.

Purpose of the interview	Description of the purpose	You have participated in an MBSR (Mindfulness-Based Stress Reduction) course for vulnerable pregnant women, which included meditations, body scans, yoga, and group discussions.<br><br>The aim of this interview is to gain a better understanding of your experience of becoming a mother, and how you use mindfulness in relation to yourself and your child.
Informed consent	Providing the consent form to the participant	I have this consent form that I would like you to sign before we begin. By signing, you agree to participate in the interview, and that your statements can be used in the study, and that the interview will be recorded. Everything will be anonymized, so your name and personal details will not appear.
Interview conditions	Time frame<br><br>Recording<br><br>Your rights and options during the interviews.	The interview will last approximately 60 minutes.<br><br>The interview will be recorded using a dictaphone. The audio file will be stored on a secure drive, accessible only to us and the Danish Center for Mindfulness.<br><br>You are very welcome to ask questions if anything is unclear during the interview. I also want to emphasize that participation is voluntary, and you may withdraw your consent at any time before the material is published. You may also choose not to answer any specific questions.

Presentation of the Participant	The participant introduces themselves	To start with, could you briefly tell us a bit about yourself? (e.g., age, partner, job/maternity leave, education)<br><br>You've now been a mother for about a year. How would you describe your child? Can you tell us a bit about who your child is? (child's age?)

**The interview**	
Theme / research question	*Interview question*
Mindfulness<br><br><br>How do participants understand mindfulness?	Opening question<br>- What do you understand by mindfulness? What does mindfulness mean to you?<br><br>Did you take anything with you from the course?<br>- If yes: Is there anything you still use from the course?<br>- If yes: How do you use it? (formal/informal practice)<br>- If no: What do you think the reason might be?
Transition to motherhood:<br><br><br>How is the transition to motherhood experienced?<br><br>Do participants experience being able to use elements from the mindfulness course in the transition to motherhood? If so, how?	**Transition**: Now that we've talked about what mindfulness is to you, the next part will be about your experience of becoming a mother and whether you've been able to use mindfulness in that context.<br><br>*Open question*<br>- How has it been to become a mother?<br>- How has it been to get to know yourself in a new role?<br><br>***Experience of becoming a mother and use of MBSR elements***<br>Have you taken anything from the mindfulness course into your experience of becoming a mother?<br>- If yes: Can you remember a situation where you used it?<br>- If no: Have you thought about how you want to be as a mother?<br>When have you experienced that you couldn't use mindfulness?<br>- **Follow-up**: Could you tell us more about that? Any examples?
Relation to the child<br><br><br>Do participants	**Transition**: We've now talked about your experience of becoming a mother and using mindfulness. Now we'd like to ask whether you've been able to use mindfulness when you're with your child.

feel able to use mindfulness elements in relation to their child? If so, how?	*Closed question:*<br>Have you experienced being able to use something from the mindfulness course with your child?<br>- If yes: Can you give a concrete example?<br>- If no: What might be the reason?<br><br>Have you been in situations with your child where you didn't feel able to use mindfulness?<br>- If yes: Can you recall a specific situation where you couldn't use it?<br>- If no: How have you used it in situations with XX? (e.g., crying, lack of sleep, low energy, night awakenings, illness, expectations of being a mother, etc.)
Optional – if not previously covered	- What made you say yes to participating in the course?<br>- Do you have prior experience with mindfulness?<br>- Have you participated in similar mindfulness courses afterwards?
Follow-up Questions from the Second Interviewer	Before we wrap up, Nanna/Luna may have a few follow-up questions, so I'll now hand it over to Nanna/Luna.

**Debriefing after the interview**		
Main Points from the Interview	The interviewer mentions some main points from the interview:	"In the interview, you mentioned…"
Feedback from the Participant	The interviewer asks if the participant has anything they would like to add:	"Is there anything more you'd like to say before we end the interview?"
Experience of the Interview	The participant shares their experience of being interviewed:	"What was it like for you to take part in this interview?"
Contact Information	The interviewer says:	"You are welcome to contact us if you need to talk about anything related to the interview or if you have further questions."
